# HOUSING AND COMMUNITY FOR OLDER ADULTS AS AGING IN PLACE (AIP) ENABLER: AN INTERNATIONAL PERSPECTIVE

**DOI:** 10.1093/geroni/igae098.1326

**Published:** 2024-12-31

**Authors:** Sojung Park, Tine Buffel

**Affiliations:** Chair; Discussant

## Abstract

This symposium discusses various approaches from five different countries that enable Aging in Place (AIP) through housing and community. The achievement of AIP depends on the institutional and cultural context. A study from New Zealand focuses on making new small homes accessible, and shows while accessible design is considered good design, not everyone understands it. User-centered accessible design is not commonly used, and trade-offs exist between accessibility, affordability, and size. A Korean study examines loneliness in older women living alone in a rural senior cohousing community. The findings show senior cohousing helps protect against loneliness by serving as a community hub for gathering, sharing, and caring. A Japanese study found “IBASHO” activities (i.e., place-based specific activities) help promote permanent community living in marginalized communities at higher risk of depopulation. Participating in such activities makes residents feel at ease in their community, even if they live alone or have poor health. A Canadian study discussed the development of a Naturally-Occurring Retirement Community (NORC) in Vancouver, formed by East Asian older adults who could not travel abroad due to border closures during the pandemic. The study explores the health-promoting nature of mutuality and its potential for population-level spillover effects on community well-being beyond the direct reach of formal programs. The last study from Hong Kong found significant improvements in perceived age-friendliness, with the greatest improvements among low-income older adults. The empirical knowledge from these studies offers valuable insights into a contextually rich understanding of a wide range of housing and communities.

